# Signification of Serum Alpha-Fetoprotein Levels in Cases of Compensated Cirrhosis and Hepatitis C Virus without Hepatocellular Carcinoma

**DOI:** 10.25122/jml-2019-0076

**Published:** 2020

**Authors:** Daniela Manuc, Carmen Monica Preda, Irina Sandra, Cristian Baicus, Razvan Cerban, Ileana Constantinescu, Andrei Ovidiu Olteanu, Cosmin Alexandru Ciora, Teodora Manuc, Daniela Elena Chiriac, Andreea Elena Chifulescu, Mircea Diculescu, Cristian Tieranu, Lucian Negreanu, Gabriela Oprea-Calin, Mircea Manuc

**Affiliations:** 1. Public Health Department, Carol Davila University of Medicine and Pharmacy, Bucharest, Romania; 2. Gastroenterology and Hepatology Department, Fundeni Clinical Institute, Carol Davila University of Medicine and Pharmacy, Bucharest, Romania; 3. Internal Medicine Department, Colentina Hospital, Carol Davila University of Medicine and Pharmacy, Bucharest, Romania; 4. Immunogenetics Department, Fundeni Clinical Institute, Carol Davila University of Medicine and Pharmacy, Bucharest, Romania; 5. Gastroenterology and Hepatology Department, Elias Emergency Hospital, Bucharest, Romania; 6. Gastroenterology and Hepatology Department, Emergency University Hospital, Carol Davila University of Medicine and Pharmacy, Bucharest, Romania

**Keywords:** Alpha-fetoprotein (AFP), hepatitis C virus, compensated liver cirrhosis, FibroMax

## Abstract

AFP (alpha-fetoprotein) levels are increased during the development of HCC (hepatocellular carcinoma); nonetheless, it can also be produced by non-tumoral hepatocytes in conditions of high cell turnover. Our study aims to provide additional data regarding the causes of elevated AFP in patients with liver cirrhosis due to hepatitis C virus (HCV) infection.

We conducted an observational prospective cohort study that included 2068 patients with compensated cirrhosis and chronic hepatitis C genotype 1b infection. The two main inclusion criteria were the presence of advanced liver fibrosis - Metavir stage F4 - diagnosed by FibroMax testing, Fibroscan or liver biopsy, and the presence of detectable HCV RNA in the serum. Plasmatic AFP levels were determined through the electrochemiluminescence method, with a standard value ranging from 0 to 7 ng/ml. All data were obtained from the Romanian National Health Agency.

The average AFP serum levels in patients with compensated cirrhosis without HCC were 9.4 ng/ml (range 0.5 ÷ 406 ng/ml); 30.1% of patients had significantly increased levels of AFP (>15 ng/ml). High values of serum AFP in patients with compensated liver cirrhosis without HCC was correlated with more advanced age (p<0.001), severe necroinflammatory activity detected by FibroMax (p<0.001), severe NASH (p<0.001), severe steatosis (p<0.001), low platelets (p<0.001), increased values of AST and ALT (p<0.001).

## Introduction

Hepatitis C virus (HCV) infection is a major player in chronic liver disorders worldwide with possible implications in long-term health status and can lead to a significant number of deaths. Hepatitis C infection is considered a public health problem, and as a result of new therapies, we are proceeding to its eradication [1, 2].

Chronic HCV infection can eventually be complicated with liver cirrhosis, hepatocellular cancer, and liver failure [3]. Recent data is showing that while HCV infection incidence has decreased in economically developed countries, the mortality rate associated with it will be rising over the following 20 years [4]. The approximate worldwide prevalence of HCV infection is 2.2%, resulting in about 130 million HCV-positive individuals. Current estimations are that HCV infection is responsible for 27% of cirrhosis cases and 25% of HCC (hepatocellular carcinoma) worldwide [5, 6].

Cohort community-based studies may represent a more significant premise for evaluating disease dynamics in a population. These suggest that for individuals infected at a younger age, the progression rate to liver cirrhosis is less than 10% within 20 years [7]. The risk of HCC in chronic HCV infection is related to the severity of fibrosis. In cirrhotic patients, the incidence of HCC is very high (1-7% per year); however, in rare cases, it can also emerge in patients with a lower degree of hepatic fibrosis [8].

Alpha-fetoprotein (AFP) is a plasma protein synthesized during the embryonic life by the gestational sac and by the fetal liver. Plasma AFP level starts to decrease before birth and is undetectable or low in healthy people. High levels of AFP in patients with HCC have been found for over 40 years [9, 10, 11]. Testing of AFP is useful in the diagnosis of hepatocellular carcinoma (HCC) only if the serum concentration of AFP is markedly elevated [12].

Certain factors may determine AFP growth, among which cirrhosis, high MELD (Model for End-stage Liver Disease) scores, and high ALT (Alanine Aminotransferase). Serum AFP levels depend on the interaction between ALT values and the development and onset of HCC. In patients who did not have HCC, the AFP level correlated with the level of ALT. Some studies plead for an existing correlation even between AFP and AST (Aspartate Aminotransferase). It seems that levels of AFP increase disproportionately to or unaccompanied by increases in levels of ALT [13, 14]. Therefore, an increased level of AFP is not necessarily associated with the presence of HCC, but can also be correlated with AST, grade III/IV of fibrosis or prolonged INR (International Normalized Ratio) [15].

AFP is no longer considered a surveillance tool for HCC; therefore, we have to block the concept that AFP is an essential instrument in diagnosis or screening, especially given its low sensitivity and specificity [16, 17].

Some studies support the fact that in patients with chronic liver disease, AFP levels can obscure many HCCs and improperly lead to HCC suspicion in many patients. Its significance is influenced by the underlying infection causing chronic liver disease. An increased AFP level may indicate HCC occurrence in patients without hepatic infections [18, 19].

Using different AFP cut-off values for HCV-positive and HCV-negative patients in order to increase AFP accuracy could represent a possible solution. Individuals without HCV infection seem to have less non-tumoral secretion of AFP; thus, even a slight increase in serum AFP might lead to the suspicion of HCC transformation. Contrarily, subjects infected with HCV often have elevated AFP levels even without HCC. Close monitoring of low-degree fluctuations in AFP levels should be recommended [20].

Apart from HCV infection, some other features can influence the sensitivity and/or specificity of AFP, including AST, HIV (Human Immunodeficiency Virus) status, or black race. The association between a higher level of AFP and AST is considered normal because AFP can be produced from non-tumoral hepatocytes in conditions of high cell turnover [21]. Also, it seems that there is a connection between the AFP level and thrombocytopenia, a phenomenon incompletely studied [22].

Consequently, the AFP value cannot be interpreted singly, and it is necessary to be correlated with imaging findings to evaluate the HCC risk in patients with a chronic hepatic disease. We propose to evaluate the conditions for increasing the AFP value in patients with compensated cirrhosis and chronic C virus infection, given that, in this case, studies claim that most of these patients do not necessarily associate HCC.

## Material and Methods

We identified adult patients diagnosed with compensated liver cirrhosis due to virus C infection that had at least one AFP test after HCV diagnosis. The database compiled multiple smaller bases from the Romanian National Health Agency.

This is a multicentric, prospective study that included 2068 patients. The inclusion criteria were as follows:

1. Age greater than 18 years old;
2. The presence of advanced liver fibrosis - Metavir stage F4 - diagnosed by Fibromax/Fibrotest testing (between 0.75 and 1.00), Fibroscan (over 14 kPa) or liver biopsy and the presence of detectable serum HCV RNA;
3. No history of hepatic decompensation, including ascites or variceal bleeding;
4. Hepatitis C virus infection with detectable HCV RNA;
5. All patients that had recent imaging examinations excluding hepatic lesions suggestive for hepatocellular carcinoma (at most three months prior to inclusion; possible examinations: abdominal ultrasound, computed tomography or magnetic resonance imaging)
6. Lack of significant ethanol consumption over the last three months.

Patients with metabolic causes of liver disease (e.g., steatosis) were not excluded.

Recorded data for the enrolled patients included age, gender, body mass index, parameters estimated by Fibromax (fibrosis stage, steatosis score, necroinflammatory activity), presence of significant comorbidities, routine blood tests (complete blood count, transaminases, total bilirubin, and INR), AFP and HCV viral load. Serum AFP levels were determined through the electrochemiluminescence immunoassay method, with a standard value ranging from 0 to 7 ng/ml. In order to define significantly increased AFP levels, we used a cut-off value of 15 ng/ml (above which investigations for HCC are recommended, according to Trevisani et al.)[18].

All patients included in the study had genotype 1b HCV infection.

FibroMax™ (Biopredictive, Paris, France) is a laboratory non-invasive procedure for liver damage evaluation through the association of three tests: FibroTest™, SteatoTest™ and NashTest™, respectively. It is excellently validated for HCV, especially for diagnosing cirrhosis (AUROC 0.87) [23].

This prospective study was approved by the National Ethics Committee of Medicines and Medical Devices. Before the beginning of the study, all patients signed written informed consent.

### Statistical analysis

We summarized ordinal and scale variables with non-normal distribution as median (min, max), and the Mann-Whitney U test was used to compare them. The categorical variables were summarized as number (%), and we used Fisher's exact test for comparison.

A two-sided P value of <0.05 was chosen for statistical hypothesis testing. Data analyses were performed using statistical software (Stata 11 from StataCorp LP, College Station, TX, USA, and SPSS version 20.0 from IBM Corporation, Armonk, NY, USA).

## Results

### Baseline demographics

Baseline characteristics for the 2068 enrolled patients are summarized in Table 1. The average age was 59 years (minimum 25, maximum 82); regarding sex, 49.2% were males, and 50.8% were females. All the patients included in the study had Caucasian ethnicity. The BMI average (body mass index) was 27.2 kg/m2. FibroMax testing showed that 1212 (58.6%) had severe necroinflammatory activity, 1014 (50.1%) had severe non-alcoholic steatohepatitis (NASH) while 1400 (67.7%) had severe steatosis. Median platelet count was slightly below the normal value (133000/mm3), and median transaminase levels showed moderate hepatocytolisis.

**Table 1:** Clinical and demographic characteristics of patients with compensated liver cirrhosis and hepatitis C virus infection.

Parameter	Percentage (%) or average (range)
**Male gender**	49.2%
**IFN Pre-treated patients (relapsers/non-responders)**	67.3% (39.1%/57.3%)
**Co-morbidities**	37.3%
**HBs-Ag positive patients**	34/2068 (1.6%)
**Class A Child Pugh score (6 points)**	216/2068 (10.44%)
**Class B Child Pugh score (7 points)**	11/2068 (0.5%)
**BMI**	27.2 (16.84÷44.9)
**Age**	60 (25÷82) years
**ALT**	88 (19 ÷ 604) IU/ml
**AST**	77 (20 ÷ 431) IU/ml
**Platelets**	133000/ mm3 (12000 ÷ 650000)
**Total bilirubin**	0,9 (0,1 ÷ 3.61) mg/dl
**INR**	1.11 (0.34 ÷3)
**MELD**	8.09 (6 ÷ 22)
**e Cl Cr**	98 (15 ÷ 347) ml/min
**Blood glucose**	105 (60 ÷ 454) mg/dl
Severe non-alcoholic steatohepatitis*	50.1%
Severe necroinflammation (grade 3 or 4 by Fibromax)*	67.5%

* estimated through FibroMax™ (Biopredictive, Paris, France)

IFN= Interferon; e Cl Cr= estimated clearance creatinine

### Elevated serum AFP and associated factors

Average serum AFP in patients with HCV infection and compensated cirrhosis without HCC was 9.4 ng/ml and ranged between 0.5 ÷ 406 ng/ml. The cohort was divided into two groups according to their AFP levels, using a cut-off value of 15 ng/ml. In 622 patients (30.1%), serum AFP levels were significantly increased (≥15 ng/ml).

Table 2 summarizes the clinical, biological, and virological parameters of the two groups and their association with AFP levels. The median age was slightly higher in patients with elevated AFP: 66 years versus 60 years. Gender distribution was similar between the two groups. FibroMax estimations revealed significant differences: in patients with normal AFP levels, severe necro-inflammatory activity was noted in 55.2% of the cases, severe NASH in 41%, and severe steatosis in 62.4%. Higher rates were reported in patients with elevated AFP: two thirds (66.6%) had severe necroinflammatory activity, severe non-alcoholic steatohepatitis was observed in 67.8%, and severe steatosis in 80.1%. Regarding biological screening, transaminase levels were above the normal range in both categories, with a median value of 2.5× upper limit of normal (ULN) in patients with elevated AFP compared to 1.5× ULN in the other group. Platelet count was also lower, with more significant thrombocytopenia (median =113000/mm3) associated with high AFP levels. The median value of total bilirubin remains within normal limits within both groups. All other biological parameters (glucose, INR, and creatinine) showed no difference between the two groups. The median viral load was also similar.

**Table 2:** Demographic and laboratory features of patients with compensated cirrhosis due to virus C infection without HCC according to their AFP serum level.

Parameter	AFP <15 ng/ml (N=1446)	AFP ≥ 15 ng/ml (N=622)	p-value
Age*	60 (25, 82)	66 (33, 82)	<0.001
**Male Sex**	719/1446 (49%)	299/622 (48)	0.055
Severe necroinflammatory activity**	798/1446 (55.2%)	414/622 (66.6%)	<0.001
Severe NASH**	593/1446 (41%)	421/622 (67.8%)	<0.001
Severe Steatosis**	902/1446 (62.4%)	498/622 (80.1%)	<0.001
Platelets (x103)*	143 (12, 1752)	113 (33, 650)	<0.001
AST*	69 (20, 338)	97 (21, 431)	<0.001
ALT*	82 (9, 604)	103 (11, 526)	<0.001
Blood glucose*	105 (60, 367)	106 (69, 454)	0.07
INR*	1.1 (0.7, 3.4)	1.1 (0.3, 2.9)	0.7
Creatinine*	0.8 (0.3, 4.3)	0.8 (0.3, 2.2)	0.1
Total Bilirubin*	0.86 (0.1, 3.47)	0.97 (0.2, 3.2)	<0.001
HCV RNA (x103) (IU/ml)*	886 (0.231, 27,795)	908 (0.24, 22,800)	0.795

* (median, range)

** estimated through FibroMax™ (Biopredictive, Paris, France)

**Figure 1:**
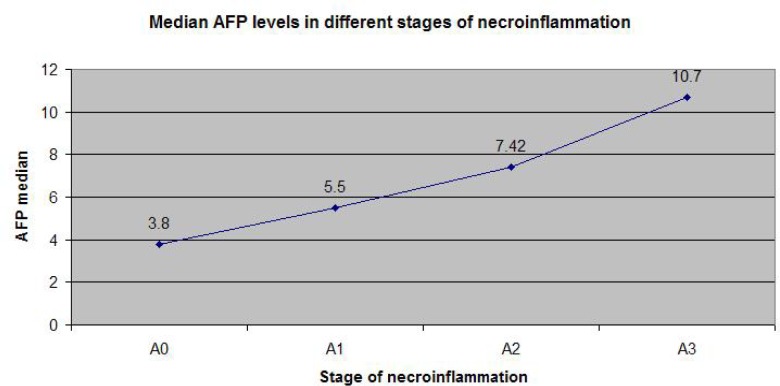
Median AFP levels in different stages of necroinflammation (according to Fibromax).

**Figure 2:**
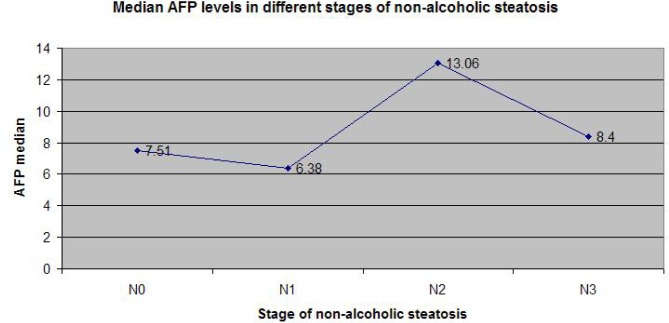
Median AFP according to the severity of nonalcoholic steatohepatitis (estimated by Fibromax).

**Figure 3:**
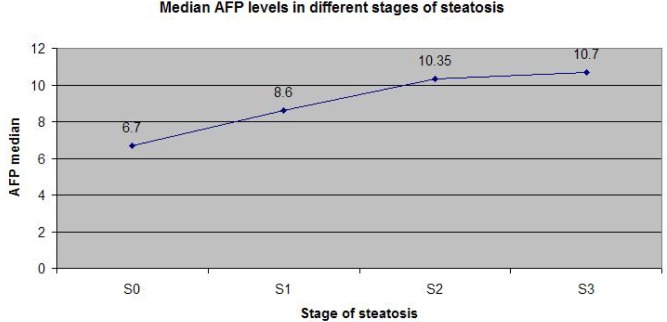
AFP levels (median) in different stages of steatosis (according to Fibromax).

Figure 1 illustrates that the most strong correlation of AFP is with the necroinflammatory activity estimated by Fibromax, while Figures 2 and 3 show that the correlation of AFP correlates weaker with the severity of steatohepatitis and with the steatosis grade.

Higher levels of AFP (over 15 ng/ml) in patients with compensated liver cirrhosis without HCC were significantly correlated with advanced age, severe necroinflammatory activity detected by FibroMax, severe NASH, severe steatosis, low platelets and increased values of AST and ALT.

## Discussions

AFP is a widely tested biomarker in HCC. The EASL (European Association for the Study of the Liver) recommends AFP usage when defining at-risk populations for HCC in case of persistently elevated levels [24]. However, its performance is suboptimal as fluctuating levels might also occur in hepatitis B or C viral flares or decompensated cirrhosis, in addition to HCC development [25].

The Romanian governmental program requested the inclusion of patients with chronic HCV and F4 fibrosis in the national cohort for the new Interferon-free antiviral therapy. As is known, this program was a great success, leading to a 96.6% sustained viral response by intention-to-treat [29]. The main advantage of our study is that it includes a high number of patients and a homogenous population: all patients had genotype 1b infection and compensated liver cirrhosis. As we previously reported, genotype 1 b is almost exclusively present in Romania (99.6% of patients) [30].

Previous epidemiologic data show that elevated AFP levels vary from 10% to 43% of people with HCV and compensated cirrhosis [26-28], depending mostly on sample size and inclusion criteria. Fattovich followed 384 patients with compensated liver cirrhosis due to HCV infection without HCC at enrollment, of which 200 patients had data regarding AFP levels. A total of 43% of patients were reported to have AFP levels above the cut-off value of 10 µg/l [26]. In another study conducted by Hu et al. on a cohort of 357 patients with different stages of virus C infection without HCC, the prevalence of elevated serum AFP (>10µg/l) was 23%, 24.5% and 42% in hepatic fibrosis stages 0-II, III and IV, respectively [15].

Chu et al. reported similar data to our results (29% of patients with elevated AFP) in a Chinese population with chronic hepatitis C, including compensated cirrhosis [28]. The same study indicates a positive correlation with age and periportal necroinflammation, which is also encountered in our study. However, Chu et al. found that the presence of steatosis was similar in the two groups of patients with and without elevated AFP.

Furthermore, in a cross-sectional study that enrolled 9800 subjects, 2601 of which diagnosed with fatty liver disease (FLD), subjects with FLD had higher serum AFP levels than those without [31], which is concordant with our results. In this Chinese study, the diagnosis of FLD was based on ultrasonography criteria, which is a different methodology. Our diagnosis of FLD was more defined since the grading of steatosis and NASH was based on Fibromax parameters, allowing a more precise correlation between AFP serum levels and FLD severity. Another Asian cohort, including 654 patients with chronic hepatitis C with no documentation of HCC and no history of liver decompensation, concluded that increased AFP levels paralleled with age, necroinflammation score, high transaminase levels and low platelet count [1]. Elevated AFP was found in 23.9% of the patients, provided a cut-off value of 6 ng/ml.

Correlations with the HCV genotype were not possible in our study due to the high prevalence of genotype 1b in the Romanian population. Hu et al. [15] and Tai et al. [1] found no significant association between HCV genotype and AFP levels, while Chu et al. proved that genotype 1b could be associated with elevated AFP. All the above-mentioned studies reported that the HCV viral load was not correlated to AFP levels, results that are similar to our findings.

Another limitation of our study is the lack of follow-up regarding liver decompensation and the development of HCC in patients with elevated AFP levels, which requires further monitoring of patients included initially.

## Conclusions

Significantly increased serum AFP values (>15 ng/ml) can be found in 30.1% of patients with compensated liver cirrhosis due to HCV infection without HCC. Increased serum AFP correlated with advanced age, severe necroinflammatory activity detected by FibroMax, severe NASH, severe steatosis, low platelets, increased values of AST, and ALT.

## Conflict of Interest

The authors confirm that there are no conflicts of interest.
